# DSM-5: a collection of psychiatrist views on the changes, controversies, and future directions

**DOI:** 10.1186/1741-7015-11-202

**Published:** 2013-09-12

**Authors:** Charles B Nemeroff, Daniel Weinberger, Michael Rutter, Harriet L MacMillan, Richard A Bryant, Simon Wessely, Dan J Stein, Carmine M Pariante, Florian Seemüller, Michael Berk, Gin S Malhi, Martin Preisig, Martin Brüne, Paul Lysaker

**Affiliations:** 1 Department of Psychiatry and Behavioral Sciences, University of Miami, Leonard M. Miller School of Medicine, Miami, FL, USA; 2 Departments of Psychiatry, Neurology, Neuroscience and The Institute of Genetic Medicine, Johns Hopkins University School of Medicine, Lieber Institute for Brain Development, 855 North Wolfe Street, Baltimore, MD 21205, USA; 3 MRC Social, Genetic and Developmental Psychiatry Centre, Institute of Psychiatry, Kings College London, De Crespigny Park, Denmark Hill, London, UK; 4 Departments of Psychiatry and Behavioural Neurosciences, and Department of Pediatrics, Offord Centre for Child Studies, McMaster University, 1280 Main St. West, Hamilton, ON, Canada; 5 School of Psychology, University of New South Wales, Sydney, NSW 2052, Australia; 6 Department of Psychological Medicine, Institute of Psychiatry, King’s College London, Weston Education Centre, Cutcombe Road, London, UK; 7 Department of Psychiatry & Mental Health, University of Cape Town and Groote Schuur Hospital, Observatory, J2, Anzio Rd, Cape Town 7925, South Africa; 8 Department of Psychological Medicine, Institute of Psychiatry, Kings College London, Room 2-055, The James Black Centre, 125 Coldharbour Lane, London, UK; 9 Department of Psychiatry and Psychotherapy, Ludwig-Maximilian-University, Nussbaumstr.7, Munich 80336, Germany; 10 IMPACT Strategic Research Centre, School of Medicine, Deakin University, Barwon Health, Ryrie Street, Geelong, VIC 3220, Australia; 11 Department of Psychiatry, Orygen Research Centre and the Florey Institute for Neuroscience and Mental Health, The University of Melbourne, Parkville, VIC 3052, Australia; 12 Discipline of Psychiatry, Sydney Medical School, University of Sydney, Sydney, Australia; 13 CADE Clinic, Department of Psychiatry, Royal North Shore Hospital, Sydney, Australia; 14 Department of Psychiatry, University Hospital of Lausanne, Site de Cery, Prilly 1008, Switzerland; 15 Division of Cognitive Neuropsychiatry and Psychiatric Preventive Medicine, LWL University Hospital, Ruhr-University Bochum, Alexandrinenstraße 1, Bochum D-44791, Germany; 16 Richard L Roudebush VA Medical Center, Indianapolis, IN, USA; 17 Department of Psychiatry, Indiana University School of Medicine, Indianapolis, IN, USA

**Keywords:** DSM-5, Psychiatry, Autism, PTSD, Mood disorders, Bipolar, Obsessive-compulsive disorders, Depression, Schizophrenia

## Abstract

The recent release of the fifth edition of the *Diagnostic and Statistical Manual of Mental Disorders* (DSM-5) by the American Psychiatric Association has led to much debate. For this forum article, we asked *BMC Medicine* Editorial Board members who are experts in the field of psychiatry to discuss their personal views on how the changes in DSM-5 might affect clinical practice in their specific areas of psychiatric medicine. This article discusses the influence the DSM-5 may have on the diagnosis and treatment of autism, trauma-related and stressor-related disorders, obsessive-compulsive and related disorders, mood disorders (including major depression and bipolar disorders), and schizophrenia spectrum disorders.

## Introduction

### The DSM-5 controversy. Tablets from Mount Sinai; a step backward or the natural progression of advances in medicine?

Charles B. Nemeroff and Daniel Weinberger

**Figure:**
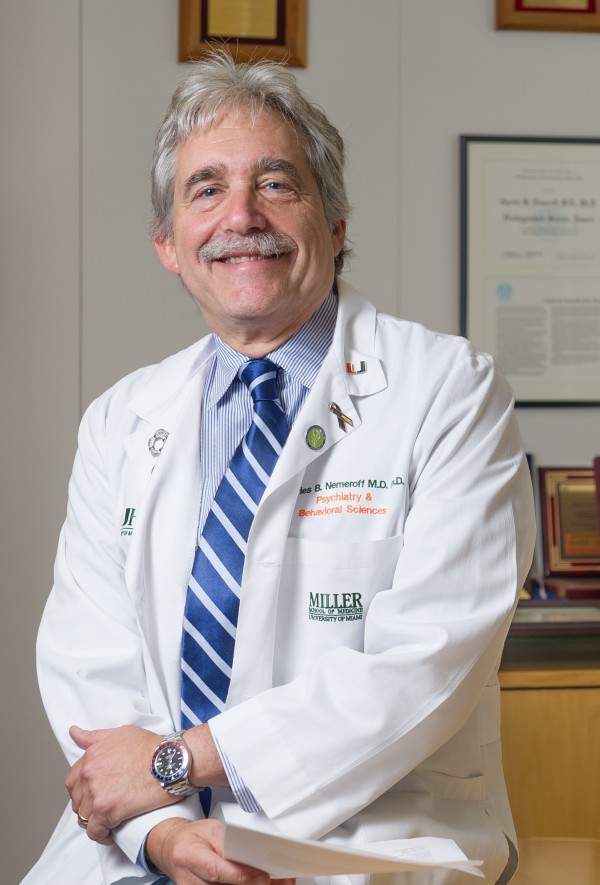
**Charles B. Nemeroff is Leonard M. Miller Professor and Chairman of the Department of Psychiatry and Behavioral Sciences at University of Miami and Director of Center on Aging at Leonard M. Miller School of Medicine.** His research interests include the biological basis of the major neuropsychiatric disorders, and he focuses on the use of genetic, neuroendocrine, neuroimaging, and neurochemical methods to comprehensively understand the pathophysiology of depression.

**Figure:**
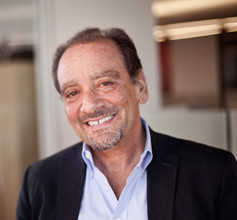
**Daniel Weinberger is Director and CEO of the Lieber Institute for Brain Development John Hopkins University School of Medicine, and Professor of Psychiatry, Neurology, and Neuroscience at the McKusick-Nathans Institute of Genetic Medicine.** His research interests include the basic neurobiological and genetic mechanisms of neuropsychiatric disorders, especially schizophrenia.

It is unlikely that when the planning for the fifth edition of the *Diagnostic and Statistical Manual of Mental Disorders* (DSM-5) [1] began 10 years ago, there was any inkling that the process and the final product would engender such a remarkable level of criticism, rhetoric, and passion as we have now witnessed. Any such large undertaking, whether it is the introduction of a new healthcare plan for a nation, or the revision of a diagnostic classification of disease, often does provoke spirited debate, but the criticism of DSM-5 from both within and outside the psychiatric community has been exceptional. Certainly, no such rancor accompanied the release of the 10th edition of the *International Classification of Disease* (ICD-10).

When the DSM-5 process was launched several years ago, the clear hope by all involved was that, finally, psychiatric diagnoses would include, in addition to signs and symptoms, various biomarkers of the major disorders including schizophrenia, bipolar disorder, and major depression, with reasonable measures of sensitivity and specificity. Because the risk for these disorders has a major genetic component, it seemed plausible to anticipate including specific genetic markers such as single nucleotide polymorphisms or structural genomic abnormalities, (for example, copy number variations), that increase disease vulnerability and perhaps denote biologically distinct alternative phenotypes. This unbridled enthusiasm followed on the heels of the sequencing of the human genome and the then-existing strong belief that many complex diseases in medicine would be simplified by the results of genome-wide association studies. However, that promise has not been realized in psychiatry, nor in many other branches of medicine, although historic insights about the genetic architecture of complex diseases have emerged. Moreover, our understanding of the underpinnings of the genetic basis of disease vulnerability and treatment response has become considerably more sophisticated because of, to name a few emerging disciplines, epigenetics, non-coding RNAs, microRNAs, transcriptomics, and proteomics.

Similar disappointments occurred in an earlier wave of unbridled enthusiasm from brain imaging studies, both structural and functional, which yielded much about the neurobiology of the major psychiatric disorders, but without any pathognomonic findings that could be incorporated into DSM-5. In retrospect, none of this is surprising, as the complexity of brain development and of the central nervous system, with its 100 billion neurons that make 500 to 800 billion connections, involves thousands of unique cell types, and influences behavior as an emergent phenomenon of interacting genetic programs and complex environmental experience. The state of the psychiatric diagnostic manual is, in reality, not much different from that of a variety of other disorders, ranging from irritable bowel syndrome, the epilepsies and dystonias, fibromyalgia, and chronic fatigue syndrome.

The challenge for any effort to revise a phenomenologically based diagnostic classification scheme is to make it better, that is, more clinically valuable and more biologically valid. It is doubtful that the current state of the art of psychiatry research has provided the tools to accomplish a major revision. Current enthusiasm for dramatic revisions based on dimensional approaches, such as the research domain criteria (RDoC), endorses the idea that variation in phenomenological characteristics of brain function, such as working memory or amygdala reactivity as measured during a functional magnetic resonance imaging (fMRI) protocol, would assign characteristics to patients that are closer to the underlying biology of their illness. The implicit assumption is that treatment would target more specifically these characteristics, rather than the syndromal classifiers. There is ample reason to be highly skeptical of this approach, at least based on the available research data. For example, patients with attention deficit hyperactivity disorder (ADHD) and patients with psychosis may show similar abnormalities of frontal lobe function, just as patients with post-traumatic stress disorder (PTSD) and patients with psychosis may show similar overactivation of the amygdala in fMRI images, but the underlying reasons for these superficial similarities are different, and so are the treatments. The treatment for frontal lobe dysfunction in ADHD (for example, stimulants) will make psychosis worse, just as the treatment for amygdala overactivation in psychosis (anti-dopaminergic drugs) is likely to have adverse effects in patients with PTSD.

In the context of the enthusiasm to revise diagnosis along the lines of biological rather than clinical phenomenology, it is sobering to recall the purpose behind the historic shift in psychiatric diagnosis represented in DSM-III. Prior to this, clinical diagnosis was based on vague, largely implicit assumptions about personality styles and psychological concepts, such as defense mechanisms. It was often noted that before DSM-III, the principal determinant of the diagnosis of a psychiatric patient was the training program of the psychiatrist. Clinicians in psychoanalytically oriented programs would interpret symptoms as indicative of specific psychological mechanisms, in contrast to trainees from a more biologically oriented program, and the resulting diagnoses would diverge, often dramatically. DSM-III introduced explicit criteria that allowed a psychiatrist on the west coast of the USA to have a much higher likelihood of reaching a similar diagnosis to a colleague on the east coast. Although the approach was never meant to establish disease validity, it rescued the field from decades of diagnostic unreliability. It is therefore potentially worrisome that classification using biological phenomena, such as working memory or amygdala responsivity, both of which are highly dependent on context and research protocols, may reintroduce some of this earlier vagueness, subjectivity, and imprecision.

Much has been made of the apparent overlap of some genes found to be associated with multiple psychiatric disorders, but the implications of these findings for a new diagnostic approach is unclear. Although the genetics of psychiatric diagnoses may show some overlap, so do the genetics of multiple sclerosis and Crohn’s disease, which are distinct clinical conditions requiring different clinical interventions. The fact that there may be shared genetic and environmental contributors to both major depression and PTSD, such as early life trauma and *FKBP5* polymorphisms, should not be surprising, and does not in any way detract from the utility of these categorical diagnoses for the practicing physician. Ultimately, a principle reason for clinical diagnosis is to predict the course of illness and to prescribe treatment. Current psychiatric medications are symptomatic treatments and although many are helpful beyond a single diagnosis, there are some dissociations of efficacy that conform to the existing diagnostic boundaries and exemplify their continuing utility. For example, lithium is not an effective antipsychotic, but it is nearly curative for many patients who fulfill the diagnostic criteria for bipolar disorder. Likewise, stimulants tend to make patients with psychosis worse, but they help most patients with an ADHD diagnosis. Clozapine, an especially effective treatment for schizophrenia, is not effective in autism, intellectual disability, ADHD, or PTSD.

Although imperfect, and we could be critical of one or another of the individual changes in DSM-5, it is the natural and appropriate evolution of the DSM based on current evidence, from the original classification system that started with DSM-I in 1952. One example of a solid improvement is the removal of the exclusion criterion for bipolar disorder if a patient ‘switched’ into a manic episode after treatment with an antidepressant. Because there is now overwhelming evidence that such ‘switches’ occur uniquely in patients with bipolar disorder and not in those without bipolar disorder, that exclusion criterion present in DSM-IV has now been removed. Similarly, the relocation of premenstrual dysphoric disorder from the Appendix to the Mood Disorders section is based on a decade of research validating this diagnosis.

So why all the controversy? Do the American Neurological Association or the American Cancer Society meetings have demonstrators outside their national meetings protesting their disease classifications? Do they have those in their ranks refusing to use their new ICD-10 disease classification? Clearly not.

Was DSM-5 handed down to our field on tablets from Mount Sinai? Of course not. Were the designers of DSM-5 intentionally anti-scientific? Of course not.

Will DSM-5 further the field by clarifying categorical disease classification? Yes.

Overall, is it an improvement over DSM-IV? Yes, but perhaps not what we all wished for at this stage in our field.

Will DSM-6 include sensitive and specific diagnostic tests that are biologically based for schizophrenia, bipolar disorder, and PTSD, to name a few? We all certainly hope so.

### Competing interests

CBN has received grants or research support from the National Institutes of Health (NIH) and Agency for Healthcare Research and Quality (AHRQ); has provided consultation for Xhale, Takeda, SK Pharma, Shire, Roche, Lilly, Allergan, and Mitsubishi Tanabe Pharma Development America; is a stockholder of CeNeRx BioPharma, PharmaNeuroBoost, Revaax Pharma, and Xhale; and has received income or equity from AstraZeneca Pharmaceuticals, PharmaNeuroBoost, CeNeRx BioPharma, NovaDel Pharma, Reevax Pharma, American Psychiatric Publishing, and Xhale. Other financial interests include CeNeRx BioPharma and PharmaNeuroBoost. CBN also holds patents for methods and devices for transdermal delivery of lithium (US 6,375,990B1) and methods of assessing antidepressant drug therapy via transport inhibition of monoamine neurotransmitters by *ex vivo* assay (US 7,148,027B2). He is also on the scientific advisory boards of American Foundation for Suicide Prevention (AFSP), CeNeRx BioPharma (2012), National Alliance for Research on Schizophrenia and Depression (NARSAD), Xhale, PharmaNeuroBoost (2012), Anxiety Disorders Association of America (ADAA), Skyland Trail, and AstraZeneca Pharmaceuticals (2009); and is on the board of directors of AFSP, Mt. Cook Pharma (2010), NovaDel (2011), Skyland Trail, Gratitude America, and ADAA.

DW has no competing interests to declare.

## Autism

### DSM-5 and autism

Michael Rutter

**Figure:**
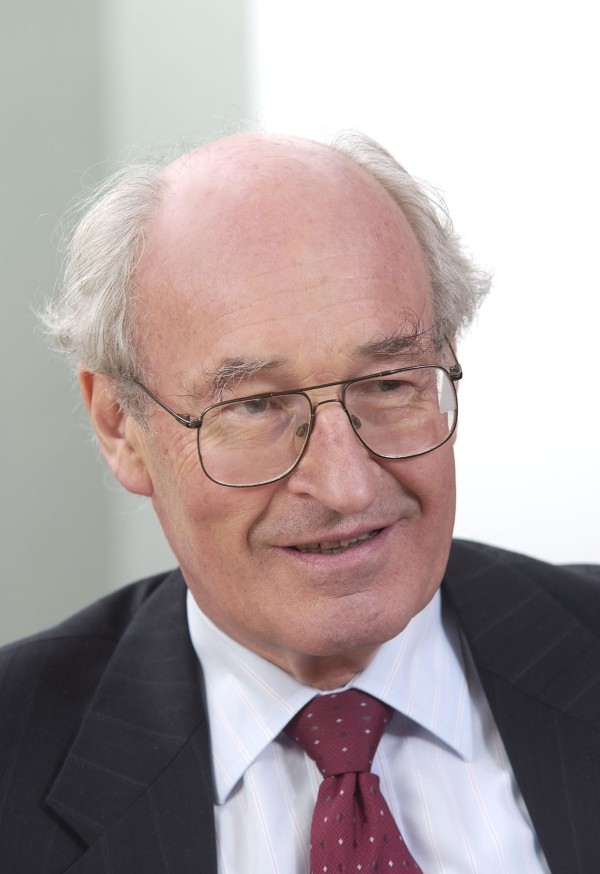
**Michael Rutter is Professor of Developmental Psychopathology at the Institute of Psychiatry, Kings College London, and Consultant Psychiatrist at the Maudsley Hospital, London.** He is founder of the Medical Research Council Child Psychiatry Research Unit and also the MRC Social, Genetic and Developmental Psychiatry Centre. His research interests include the genetics of autism, the development of diagnostic and screening measures for autism, antisocial behavior, studies of both school and family influences on children’s behavior, and the interplay between genetic and psychosocial risk factors.

Overall, DSM-5 [1] is disappointing because it failed to deal with many of the serious challenges in relation to DSM-IV. There has been a very marginal decrease in the total number of categories, but the number is still far too many for any clinician to remember the criteria for each. The co-occurrence of diagnoses (misleadingly termed ‘co-morbidity’) is likely to remain unacceptably high. No notice has been taken of the growing evidence of the lack of distinctiveness between diagnoses. Because the diagnoses requiring further testing have been placed in an appendix, this means that their systematic testing will be impossible (or at least very difficult). It has been said that as new research findings relevant to classification become available, these will lead to modifications in the classification, but who will decide when and how this is necessary? The desirability of harmonization with ICD-11 has been almost entirely ignored, and it is clear that financial considerations have meant that the two major classifications will remain very different. What has governed the decisions on changes? It would have been reasonable to consider clinical utility, but it seems that this has had a low priority. It could have been scientific validity but this was examined only in a very partial way with respect to individual proposals from working groups, rather than in relation to DSM-5 as a whole.

What about autism spectrum disorders (ASD)? Clearly, it was necessary to abandon the sub-classification in DSM-IV, because it had been ignored or sidestepped by most clinicians and researchers. Despite the plausibility of the sub-divisions, they had not proven workable, and it was right to abandon them. Will this lead to a substantial number of individuals with ASD now failing to meet the criteria for diagnosis? We do not know, but it is a legitimate concern, especially with high-functioning individuals. What about the loss of the categories of disintegrative disorder and Rett syndrome? The former deals with an uncommon diagnosis of uncertain validity, but the possibility of testing that has been lost through elimination of the diagnosis. This removal is in contrast to ICD-11, in which it is likely that it will be specified as a diagnosis requiring further testing.

The effects of dropping the coding of Rett’s syndrome are rather different. Rett’s syndrome has been shown to be a valid diagnosis and, thus, including it here as a specifier makes no clinical or scientific sense. The presence of autistic features in an individual with Rett’s syndrome in no way affects the implications of the diagnosis of Rett’s syndrome, so why code it? The answer lies in the old-fashioned adherence to a purely descriptive classification, and the absence of any medical classification in DSM-5.

It should also be noted that the diagnosis of autism is based solely on two main domains of features, and no attention is paid to the very common period of transitory regression, the presence of savant skills, the frequent increase in head size during the pre-school years, and the frequent development of epileptic seizures, usually beginning in later adolescence/early adult life.

Clearly, obliteration of the sub-classification of ASD was needed because the existing scheme did not work, and had been ignored by most clinicians and researchers. However, it is already certain that ASD will prove to be heterogeneous (clearly so with respect to genetics, but probably also so with respect to biology). Thus, ASD will have to be sub-divided again in the future, but how best to do so remains to be established.

In short, I do not know how DSM-5 will affect the diagnosis of autism. Probably not very much, but because DSM-5 will affect access to services, there is a legitimate concern that some individuals (probably those with higher-functioning autism) may lose out. What is most obvious is that DSM-5 has completely ignored the reality of the broader phenotype of autism. There is abundant evidence that this phenotype exists, despite the continuing lack of good criteria for diagnosis. Given the importance of this phenotype for the possibility of early intervention, this is a really important opportunity that has been missed. It is also surprising to see no mention of the pattern of quasi-autism often seen in patients after profound institutional deprivation.

### Competing interests

From the 1960s up to early 2013 and mid-2011 respectively, MR has served on various committees concerned with ICD or DSM or the harmonization of the two. This paper, however, presents a personal view and should not be interpreted as reflecting the views of either the WHO or the APA. MR has no financial competing interests.

## Trauma-related and Stressor-related Disorders

### What’s new in DSM-5 for clinicians working with children exposed to trauma?

Harriet L. MacMillan

**Figure:**
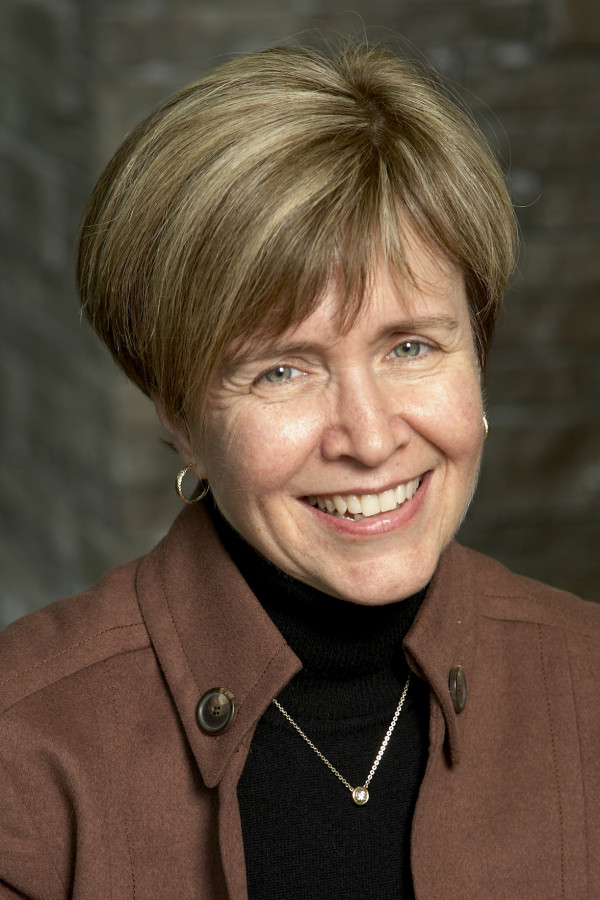
**Harriet L. MacMillan is Professor in the Departments of Psychiatry and Behavioural Neurosciences and in the Department of Pediatrics, Chedoke Health Chair in Child Studies and Acting Director of Offord Centre for Child Studies at McMaster University and McMaster Children’s Hospital/Hamilton Health Sciences.** She is a pediatrician and child psychiatrist, and her research interests include research into family violence and trials of interventions aimed at prevention of child maltreatment and intimate partner violence. She is supported by the Chedoke Health Chair in Child Psychiatry.

As a child psychiatrist and pediatrician practicing in the area of family violence, I welcome the increased focus on trauma-related and stressor-related disorders in DSM-5, which is reflected by several important changes. My hope is that these changes, as discussed below, will lead to increased recognition and understanding of the impairment associated with childhood adversities, especially in children. Ideally, this should occur not only among child psychiatrists, but among the full range of clinicians engaged in the assessment and provision of services to children, including family physicians, nurses, pediatricians, and psychologists. This aim is consistent with the view of the DSM-5 Task Force regarding the potential value of the DSM classification system to all professionals concerned with mental health care [1], not just psychiatrists. I am cautiously optimistic that the DSM-5 will now become more relevant to those of us seeing children and adolescents with stressor-related disorders across disciplines.

The key changes for those of us working in the family violence field are as follows [2]. First, the move of PTSD and acute stress disorder away from Anxiety Disorders to a new stand-alone chapter Trauma- and Stressor-Related Disorders (TSRD), reflects the current deeper understanding of the heterogeneous symptom presentation of stress-related conditions. In addition, the three major PTSD symptom clusters in DSM-IV (re-experiencing, avoidance/numbing, and arousal) have been revised to four clusters: avoidance/numbing is now divided into the two clusters of avoidance and persistent negative changes in both cognition and mood. The latter has been broadened to include negative emotional states. It is increasingly recognized that PTSD symptoms go beyond fear-based anxiety, and include dysphoria, aggression, guilt, and shame [3]. This is also the case for adjustment disorders, which are now included in the same chapter. Another important difference for the PTSD criteria in DSM-5 is the removal of the subjection reaction (intense fear, helplessness, or horror) of how an individual experienced a traumatic event.

It is encouraging to see the development of a new PTSD sub-type in DSM-5: Post-traumatic Stress Disorder for Children 6 Years and Younger. The DSM-IV PTSD criteria did not take into account the variation in symptom presentation during development, especially in young children. For example, the previous requirement for three avoidance/numbing symptoms in young children, whose capacities to verbally express such experiences are only emerging, led to under-recognition of PTSD in children [4]. The criteria now include one or more symptoms representing either persistent avoidance or negative alterations in cognitions and mood, and presence of one or more intrusive symptoms, in addition to two or more symptoms of alterations in arousal and reactivity.

The inclusion of reactive attachment disorder and a new condition, disinhibited social engagement disorder, in the TSRD chapter emphasizes the recognition of symptoms associated with neglect in childhood. In DSM-IV, reactive attachment disorder had two sub-types, emotionally/withdrawn and indiscriminately social/disinhibited, but the latter has now become a distinct disorder, based on differences in symptom presentation. Typically classification of stressor-related disorders in childhood has emphasized the impairment associated with abuse in childhood (for example, sexual abuse), rather than neglect. Both types of symptoms and their association with neglect are important to recognize.

The effects that such changes in DSM-5 will have on clinical practice are yet to be determined, but there is the potential for improved assessment of stressor-related conditions in children, now that the criteria are more relevant and appropriate. This is essential, both for identifying stressors such as one or more types of child maltreatment, which can then potentially be stopped from continuing or recurring, and for providing evidence-based interventions to reduce impairment. Increasingly we have approaches such as trauma-focused cognitive behavior therapy [5,6] and child–parent psychotherapy [7,8], which have shown benefits in reduction of PTSD symptoms in children exposed to sexual abuse and intimate partner violence, although additional trials are needed to determine the generalizability of these programs.

Critics of the DSM-5 suggest that lowering the threshold for certain conditions or expanding the symptom criteria may lead to overdiagnosis, that is, identification of conditions that do not necessarily need treatment. In the area of trauma-related and stressor-related disorders, especially in childhood, the problem has been one of underdiagnosis rather than overdiagnosis. Furthermore, the need for both a history of exposure and specific trauma symptoms, as well as association with significant distress or impairment, reduces the likelihood that overdiagnosis will occur in this area.

Perhaps the way in which the DSM-5 changes have the greatest likelihood of improving clinical care for children with stressor-related conditions is through improvement in assessment of outcomes in research evaluating the effectiveness of interventions. A recent comparative effectiveness review of interventions addressing child maltreatment [9] determined that although some programs show promise in improving child well-being and child welfare benefits, the evidence is still very limited. One of the limitations identified in this review was the ‘wide heterogeneity in type and psychometric soundness of outcome measurement across studies.’ Possibly the revised classification in DSM-5 will facilitate better outcome measures of stressor-related conditions that can then be used in ensuring high-quality evaluations of existing and forthcoming interventions.

### Competing interests

HLM has no competing interests to declare.

### Post-traumatic stress disorder in DSM-5

Richard A. Bryant and Simon Wessely

**Figure:**
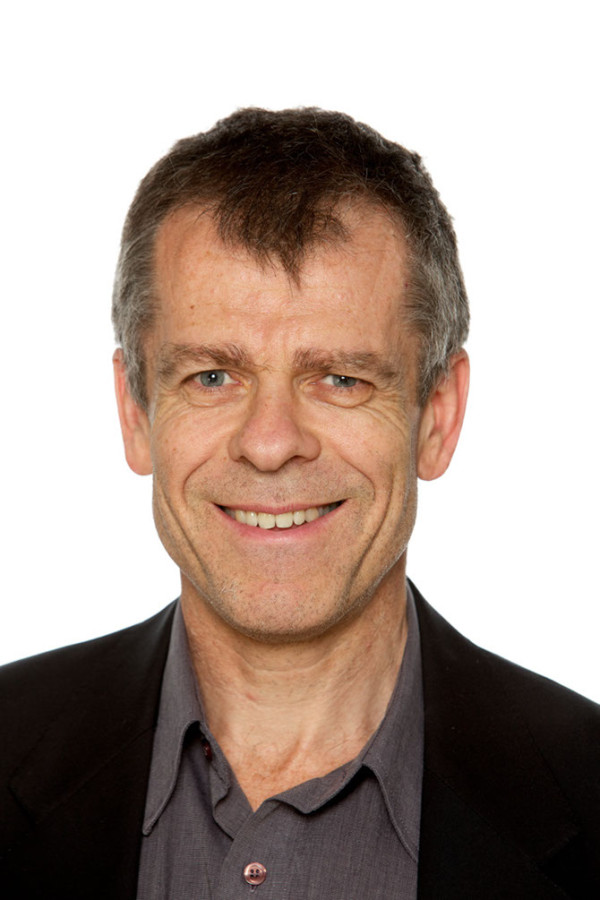
**Richard Bryant is Scientia Professor at School of Psychology, University of New South Wales.** His research interests include assessment, mechanisms, and treatment of post-traumatic stress disorder and anxiety. He served on the DSM-5 PTSD/Trauma/Dissociative Work Group and the ICD-11 Working Party on Stress-related Disorders.

**Figure:**
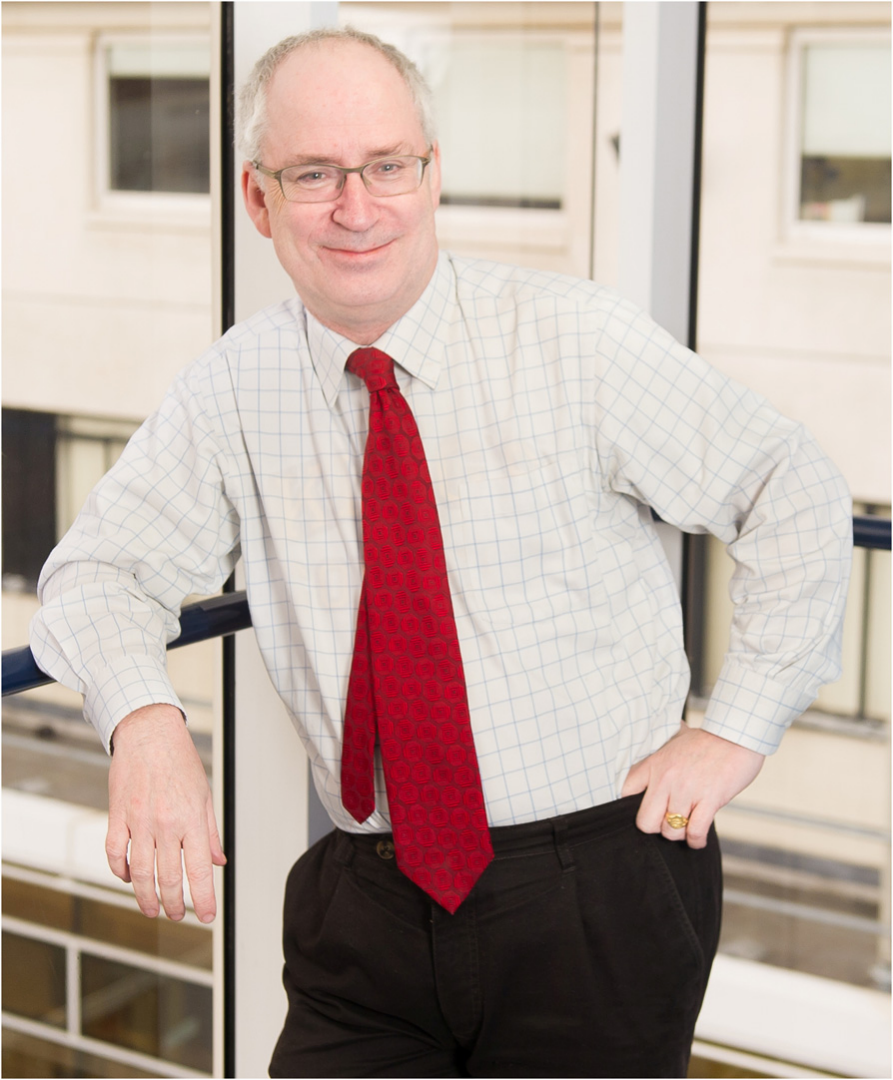
**Simon Wessely is Consultant Psychiatrist at King’s College Hospital and the Maudsley Hospital, and Professor of Psychological Medicine at Kings College London, and Vice Dean at Institute of Psychiatry, Kings College London.** His current research interests include clinical epidemiology, psychiatric injury, and military health. He also served on the ICD-11 Working Party on Stress related Disorders.

The American Psychiatric Association (APA) recently launched its latest version of DSM-5 [1] commonly known as the ‘bible’ of psychiatric disorders in many countries. One focus of the new edition has been the restructuring of the diagnostic definition of PTSD. Introduced in the 1980 third edition of DSM, and considered as either an attempt to describe a new syndrome encompassing the enduring mental-health consequences of war [10], or according to others, as the USA’s attempt to come to terms with the wider societal traumas of the Vietnam War [11], it has traditionally been conceptualized as a fear disorder defined by three clusters of symptoms: 1) re-experiencing of fear memories (for example, intrusive memories), 2) avoidance of trauma reminders (for example, amnesia, withdrawal, avoidance of situational reminders), and 3) hyperarousal symptoms (for example, sleep disturbance, heightened startle response).

The DSM-5 took a different approach, and explicitly aimed to expand the definition of PTSD beyond the fear construct. A primary motivation for this change was the perception that the term needed to accommodate trauma survivors who display symptoms of distress but who may not meet the traditional criteria for PTSD [3]. The argument was made that war veterans and victims of crime (to name just two groups of survivors) often present for clinical assistance with symptoms that are dominated by anger, guilt, or shame. Accordingly, it was felt that this should be recognized by adding an additional cluster of symptoms that formally acknowledged the role of ‘negative alterations in cognition and mood’, and included the new symptoms of pervasive negative emotional states, negative appraisals about oneself or the world, and excessive blame. Some symptoms previously noted as ‘avoidance’ symptoms (such as withdrawal and amnesia) were also included in this cluster, because of much evidence from factor analytic studies that they are distinct from active avoidance [12]. In total, DSM-5 increased the number of symptoms from 17 in DSM-IV to 20 in DSM-5. To meet the DSM-5 criteria, the individual needs to have experienced a marked traumatic experience and satisfy each of the symptoms of re-experience, avoidance, arousal, and negative alterations in mood and cognition. The person needs to have impairment, and the symptoms need to persist for at least 1 month after the traumatic event.

Do these changes represent a step forward or backward? In terms of clinical intervention, there is no doubt that the symptom composition of the new diagnosis may lead to the diagnosis being applicable to more of those exposed to trauma. If this were the case, then an increase in prevalence rates of PTSD would be expected using the DSM-5 criteria relative to the DSM-IV criteria. In fact, the limited evidence available suggests that the prevalence rate does not increase [13], which on one level is a good development because of the considerable concerns expressed in many quarters about ‘bracket creep’ with new psychiatric diagnoses; however, it poses questions concerning the extent to which the new definition has actually improved identification of those who have a post-traumatic stress condition. Many studies attest to people with sub-syndromal PTSD having comparable levels of impairment to those with full PTSD [14], emphasizing the conclusion that the arbitrary cut-off point set by the diagnostic threshold does not perform optimally in classifying those with PTSD-related difficulties.

One consequence of the new definition is that the disorder is much more heterogeneous than it was previously deemed to be. As a result of its multiple cluster format, PTSD has traditionally been a very heterogeneous disorder in DSM, because to satisfy the criteria there must be a minimum number of symptoms from each cluster: in DSM-5, at least one of five re-experiencing symptoms, one of two avoidance symptoms, three of seven cognition and mood symptoms, and three of six hyperarousal symptoms. Considering the various combinations of symptoms required to meet the definition of the one disorder called PTSD, it involves a very high number of very distinct possible combinations. In DSM-IV, this resulted in a total of 79,794 symptom presentations. By adding more symptoms and an additional cluster, DSM-5 results in 636,120 possible combinations [15]. This is in marked contrast to the conditions such as Panic Disorder (3), Social Phobia (1), Obsessive-Compulsive Disorder (3), or Major Depression (227). A potentially significant consequence of this lack of homogeneity is that it may represent a significant obstacle to research that attempts to identify characteristic markers of the disorder. Although there has been enormous attention given in recent years to biological or cognitive markers of PSTD, this research has typically resulted in mixed findings, lack of replication, or poor specificity. One strong possibility for the lack of progress on this front is that the many different clinical presentations that are encompassed by the PTSD diagnosis may hinder any attempt to identify a single mechanism or marker.

Although DSM-5 aims to enhance clinical utility, and in this sense the PTSD diagnosis tries to make it more relevant to those affected by war and crime, future studies will shed light on whether this has been successful. It is noticeable that the new proposals for PTSD in ICD-11 have taken an opposite perspective, and elected to simplify the diagnosis, while directing clinicians to core features of the disorder [16]. The increased heterogeneity reflected in DSM-5 raises serious questions about the utility of the diagnosis for furthering the research agenda. Given the many questions that abound concerning the neural, genetic, cognitive, and behavioral features of the disorder, we are doubtful that the new definition will facilitate clarification of these issues.

### Competing interests

RB has served on the DSM-5 PTSD/Trauma/Dissociative Work Group and the ICD-11 Working Party on Stress related Disorders. SW has served on the ICD-11 Working Party on Stress related Disorders. These comments reflect the opinions of the authors, and are not necessarily those of the APA, the DSM-5 Work Group, or the ICD 11 Working Party. Neither author has any other competing interests to declare.

## Obsessive-compulsive and Related Disorders

### DSM-5 and obsessive-compulsive and related disorders

Dan J. Stein

**Figure:**
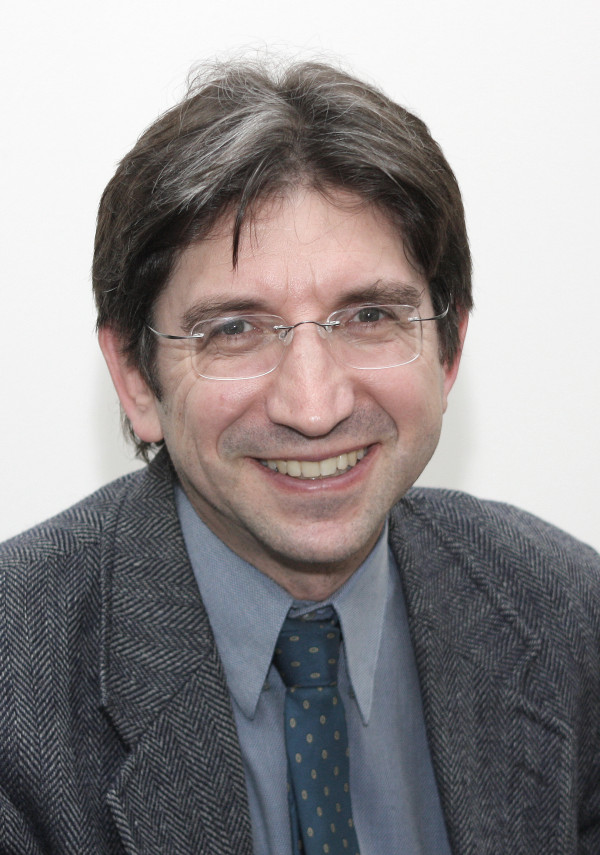
**Dan J. Stein is Professor and Chair of the Dept of Psychiatry and Mental Health at the University of Cape Town, and Director of the MRC Unit on Anxiety and Stress Disorders.** His research interests include the psychobiology and management of the anxiety, obsessive-compulsive and related disorders, and traumatic and stress disorders. He also served as Chair of the DSM-5 Sub-work Group on Obsessive-Compulsive Spectrum Disorders.

DSM-5 includes a number of changes with respect to obsessive-compulsive disorder and related conditions (OCRDs) [1]. First, the chapter on OCRDs is an entirely new one, and includes obsessive-compulsive disorder (OCD), body dysmorphic disorder (BDD), and trichotillomania (hair-pulling disorder). Second, the chapter includes several new conditions, including hoarding disorder and excoriation (skin-picking) disorder. Third, some of the disorders, such as trichotillomania (hair-pulling disorder), have new names. Fourth, there are a number of new approaches to the sub-typing of OCRDs. Fifth, there are a number of changes to the diagnostic criteria of conditions now classified as OCRDs. Finally, the text relating to OCRDs has been revised.

These changes were based on a range of scientific data including those published in the form of a research conference [17], systematic reviews [18-25], secondary analyses of existing datasets [26], and DSM-5 field surveys [27-29]. Input was also obtained from members of the OCRD clinician and research community, consumer advocates, and the professional and lay public at large [30].

Recommendations made by the DSM Sub-work Group on Obsessive-Compulsive Spectrum Disorders were reviewed by a scientific review committee, in some cases by a clinical and public health committee, and by the DSM-5 taskforce. This piece briefly reviews the implications for clinical practice.

The new chapter on OCRDs reflects growing data that OCD differs from the anxiety disorders on a number of diagnostic validators, and that there are important phenomenological and psychobiological overlaps between OCD and a number of related conditions [19]. At the same time, it is important to emphasize that there are both strong overlaps between OCD and the anxiety disorders, and important differences between the OCRDs. The close relationship between OCD and the anxiety disorders is reflected in the order of the chapters in DSM-5, and the differences between the OCRDs are emphasized in the DSM-5 text [31].

The potential advantage for clinical practice of a new chapter is that clinicians and the public will be made more aware of these underdiagnosed and undertreated conditions. The hope is that clinicians will screen more rigorously for these conditions, and that researchers will use structured diagnostic interviews and standardized symptom measures to investigate and evaluate the full range of these often-neglected conditions in a systematic way. A potential disadvantage of having a single chapter is that some clinicians will fail to appreciate the important differences that exist between these conditions.

The DSM-5 chapter on OCRDs includes several new diagnoses, namely hoarding disorder, excoriation (skin-picking) disorder, substance/medication-induced OCRD, OCRD due to another medical disorder, and other specified OCRD. The inclusion of hoarding disorder and excoriation disorder reflects a growing database on the diagnostic validity of these conditions, and the clinical utility of recognizing them in the nosology [23,32]. The inclusion of substance/medication-induced OCRD, OCRD due to another medical disorder, and other specified OCRDs is a logical consequence of having a separate chapter on OCRD.

Definitions of psychiatric disorder continue to be debated, and it is surely important not to medicalize problems of daily living [33]. However, those who meet diagnostic criteria for these conditions experience distress or impairment, and deserve appropriate intervention. The category of other specified OCRD brings attention to those individuals, who do not meet the diagnostic criteria for a DSM-5 mental disorder, but who nevertheless experience clinical distress or impairment; this category includes individuals with olfactory reference syndrome, obsessional jealousy, and body-focused repetitive behavioral disorders other than trichotillomania (hair-pulling disorder) and excoriation (skin-picking) disorder.

The name ‘trichotillomania’ has been felt by some consumer advocates to be inappropriate and confusing, as mania is not a defining symptom of this condition. While some consumer advocates have expressed preference for a plain language term such as ‘hair-pulling disorder’, others have felt that such a term would not have sufficient medical gravitas. Furthermore, there would not be continuity with the previous literature. Thus, the name ‘trichotillomania (hair-pulling disorder)’ was proposed, allowing individuals the choice of which name to use. A similar set of considerations led to a proposal for the name ‘excoriation (skin-picking) disorder’.

In DSM-5, OCD, BDD, and hoarding disorder have an insight specifier that ranges from ‘good insight’ through ‘fair insight’ and on to ‘no insight/delusional’ [34,35]. This sub-typing reflects the dimensional nature of insight in OCRDs, the existence of data indicating that different levels of insight require different treatment approaches, and a scientific consensus that patients with an OCRD that reaches delusional levels should be diagnosed with an OCRD rather than delusional disorder. In DSM-5, OCD can be specified as tic-related, BDD can be specified with muscle dysmorphia, and hoarding disorder can be specified with excessive acquisition, again reflecting the data that such specifiers have clinical implications [18,21,22].

Several changes to the diagnostic criteria for OCRDs have been made. It is notable that some of these changes help to emphasize the overlapping features of conditions in the OCRD chapter. Thus, BDD includes a new diagnostic criterion, which emphasizes that individuals respond to their appearance preoccupations with repetitive behaviors or mental state or acts. Similarly, the criteria for trichotillomania (hair-pulling disorder) no longer include diagnostic criteria that conform to the pattern of impulse control disorders, but include an extra criterion, which emphasizes that individuals have attempted to decrease or stop the hair-pulling. The validity of such changes have been confirmed by secondary analyses and field surveys [23,28].

The updated DSM-5 text on OCRDs includes new information that has emerged since the publication of DSM-IV, and provides details on key clinical issues such as prevalence, development, and course, risk and prognostic factors, culture-related and gender-related diagnostic issues, suicide risk, and functional consequences. For example, the section on OCD describes the main obsessive-compulsive symptom dimensions that have emerged from studies around the world over the past two decades [36]. Differential diagnosis of OCRDs is not always straightforward, and the text may usefully guide clinicians towards a correct diagnosis.

This brief piece does not allow a comprehensive discussion of all of the changes made in the DSM-5 chapter on OCRDs. Although any particular change to the nosology has both pros and cons [37,38], the rationale for many of the changes is provided in systematic reviews and proposals published by the DSM-5 Sub-work Group, which has attempted to take an evidence-based approach, making changes that are based on accumulating data on diagnostic validity and clinical utility. The hope is that such changes will help clinicians to recognize individuals with OCRDs, and to diagnose them appropriately. Such recognition and diagnosis may in turn lead to better treatments and improved outcomes.

### Competing interests

DJS served as Chair of the DSM-5 Sub-work Group on obsessive-compulsive spectrum disorders. He has received research grants and/or consultancy honoraria from Abbott, AstraZeneca, Biocodex, Eli Lilly, GlaxoSmithKline, Jazz Pharmaceuticals, Johnson & Johnson, Lundbeck, Orion, Pfizer, Pharmacia, Roche, Servier, Solvay, Sumitomo, Takeda, Tikvah, and Wyeth.

## Mood Disorders

### DSM-5 and depressive disorders: yet more ado about nothing?

Carmine M. Pariante

**Figure:**
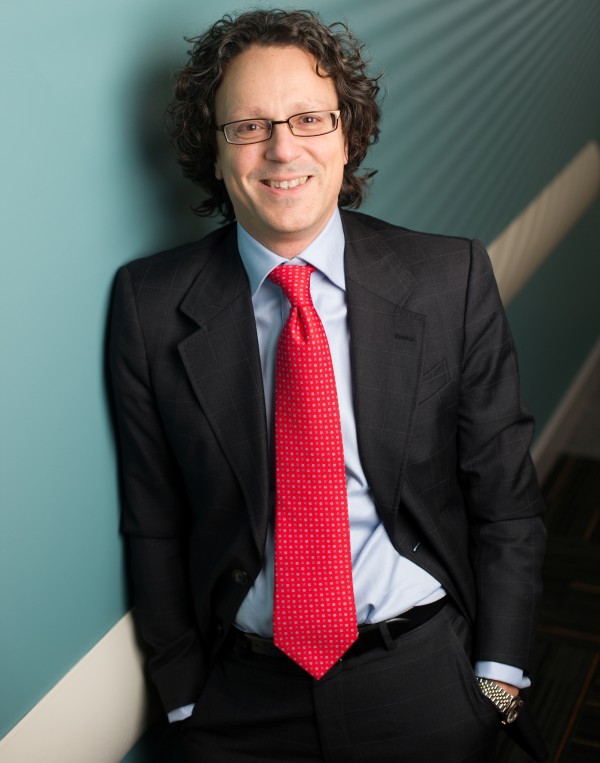
**Carmine M. Pariante is Professor of Biological Psychiatry at Institute of Psychiatry, Kings College London.** His research focuses on the biological effects of stress, the pathogenesis of depression, and the molecular mechanisms of antidepressant drugs.

Critics have accused the new DSM-5 [1] of multiplying the number of psychiatric diagnoses and of medicalizing normal human experience. Two changes in the clinical area of Depressive Disorders have been used as examples to support this accusation: the removal of the ‘bereavement exclusion’, and the introduction of the new Disruptive Mood Dysregulation disorder (DMDD) for use in children. In both cases, the accusation is wrong.

The removal of bereavement exclusion will allow individuals who have been clinically depressed for less than 2 months after the loss of a loved one to be diagnosed with Major Depression. In the previous (fourth) edition of the DSM, the diagnosis of major depression could be made only if the depression had persisted for at least 2 months after the bereavement.

Does this mean that hordes of individuals who have just lost their partner or their parent will be started on antidepressants? Obviously not: first, because individuals will still need to fulfill the diagnostic criteria for depression, including the impairment in important areas of functioning such as in their social or professional life; and second – and crucially – because no clinically competent doctor would do so. Two scenarios clarify this.

Scenario 1. A young woman loses her husband in a car crash. She has no history of psychiatric disorders. She is grief-stricken and has to take time off work. Because of the removal of the bereavement exclusion, after 2 weeks, she clinically fulfills the ‘new’ criteria for major depression, . She consults her primary care doctor, who reassures her that she is going through a very difficult process but that this is normal, and advises her to mobilize the support of her friends and family and that she will feel better in time.

Scenario 2. A young woman loses her husband in a car crash. She has had several previous episodes of severe depression, which have lasted for 1 year or more. During these previous periods of depression, she has not been able to hold on to a job, and has tried to commit suicide. Like the woman in Scenario 1, this woman is grief-stricken, has to take time off work, and after 2 weeks, clinically fulfills the ‘new’ criteria for major depression. She sees her primary care doctor, who is aware of the research evidence showing the similarities between bereavement-related depression and other forms of depression [39], and is therefore rightly concerned that this stressful event will precipitate yet another severe episode in this predisposed woman. Until recently, the clinical management of such patients within the American health system would have been influenced by the ‘bereavement exclusion’, which would have complicated the reimbursement from the medical insurances companies. However, no longer impinged by this exclusion, the doctor immediately starts the patient on treatment with an antidepressant that has proven effective for the patient in the past, and this prevents the repetition of a downward spiral.

Hence, clinical competency and personalized decisions are, as always, key to clinical management, and diagnostic textbooks will not make good doctors take bad decisions.

What about the disruptive mood dysregulation disorder? Accused of being a diagnosis for ‘grumpy children’, this disorder requires ‘persistent irritability and frequent episodes of behavior outbursts, three or more times a week, for more than a year’ to be present in order to make the diagnosis. Is this a situation that any sensible parents (let alone doctors) would call a ‘grumpy child’? I am sure that many parents would ask for help if they had a child with such difficulties.

Moreover, the criticism is particularly misplaced (and ironic) because DSM-5 has introduced this new disorder to prevent the (ab)use of the diagnosis of bipolar disorder in children. The rates of bipolar disorder in children have grown almost six-fold between 1996 and 2004 [40], and this diagnosis is accompanied by the premature use of antipsychotics and mood stabilizers in very young individuals, and by a much more stigmatizing label, especially in a child. Perhaps sometimes a new disorder is a good thing?

### Competing interests

CP receives research funding from the Medical Research Council and other governmental agencies, the National Institute of Health Research and other NHS-related funding, the Wellcome Trust and other charities, and pharmaceutical companies.

### Elimination of the major depression bereavement exclusion in DSM-5

Florian Seemüller

**Figure:**
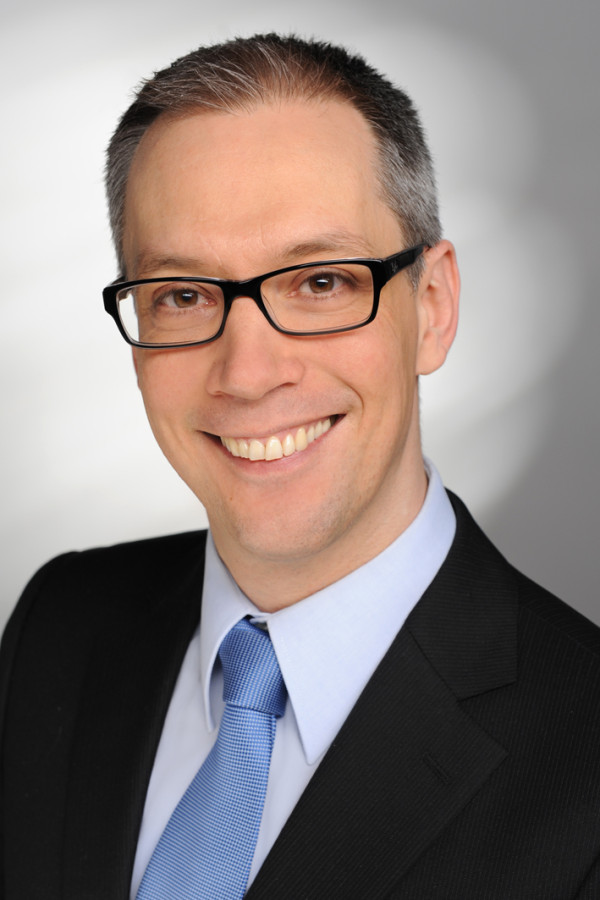
**Florian Seemüller is consultant for psychiatry at the Department of Psychiatry at the Ludwig-Maximilians-University Munich.** His research interests focuses on suicide research, bipolar disorder, and the systematic, course-related, and clinically oriented evaluation of longitudinal data from patients with depressive disorders and disorders among patients with schizophrenia.

Major depression is the worldwide leading cause of disability in terms of total years lost due to the disability. Correct diagnosis is a prerequisite for the early and correct treatment of this disorder. The DSM-5 field trials have shown that the DSM-IV diagnostic criteria for major depression have a very disappointing test-retest reliability. Although some diagnoses, such as PTSD and major neurocognitive disorder had a very good test-retest reliability (κ values of 0.67 and 0.78), and others such as bipolar I disorder (κ = 0.56), schizoaffective disorder (κ = 0.50), mild neurocognitive disorder (κ = 0.48), alcohol use disorder (κ = 0.40) and binge eating disorder (κ = 0.56) still showed good reliability, the test-retest reliability was questionable for conditions such as generalized anxiety disorder (κ = 0.20), mixed anxiety-depressive disorder (κ = −0.004) obsessive-compulsive personality disorder κ = 0.31) antisocial personality disorder (κ = 0.21) and major depression (κ = 0.28) [41].

Therefore, in order to improve the test-retest reliability and to reduce the number of false positives, tightening of the diagnostic criteria for such problem diagnoses would have been desirable; however, with the elimination of the major depression bereavement exclusion in the DSM-5 [1], the diagnostic boundaries have again been widened. Thus, a major depressive episode can be diagnosed if a person grieves for a loved one for more than 2 weeks. This challenges the view that grief after the loss of a loved one, which frequently comprises depressive symptoms, belongs to the category of healthy psychic reactions and coping strategies. This has been the reason why bereavement has been an exclusion criterion (Criterion E) for the diagnosis of major depression since DSM-III. The bereavement exclusion criterion further specified that a bereavement-related depressive episode will not be excluded as major depression and should be diagnosed as such if it lasts longer than 2 months, or if the patient displays any one of the several symptoms suggestive of severe depression, including suicidal ideation and psychomotor retardation. Grief usually resolves in about 80% of all cases after a couple of weeks or months.

Clinical research further suggests that the risk for recurrent depression in people experiencing severe grief is not different from that of healthy controls [42]. Although some individuals, especially elderly people with complicated grief, may benefit from this change, possibly by earlier receipt of intensive treatment after having lost a loved one, millions of other people might be unnecessarily labeled as having an illness, and consequently receive treatment that they do not need. Because both human and financial resources in mental health care are restricted, such broadening of diagnostic boundaries always poses the risk that patients with more severe mental illness who are in need of more and expensive treatments do not receive adequate care. In addition, this removal of the bereavement exclusion means that clinicians are not required to distinguish between normal grief and major depression any longer, and thus it may mean that this skill will soon disappear from the clinical training of psychiatry.

### Competing interests

FS has received grants and research support from Lilly, Astra Zeneca, and Glaxo-Smith Kline. He has been on the speakership bureaus of Lundbeck, Bristol-Meyers Squibb, Lilly and Astra Zeneca, Bial, BMS, Cephalon, Eli Lilly, Glaxo-Smith Kline, Janssen Cilag, Organon, Pfizer Inc, Sanofi -Aventis, Servier, UBC, and UCB Belgium.

### Take (DSM) five: jazzing up the blues?

Michael Berk and Gin S Malhi

**Figure:**
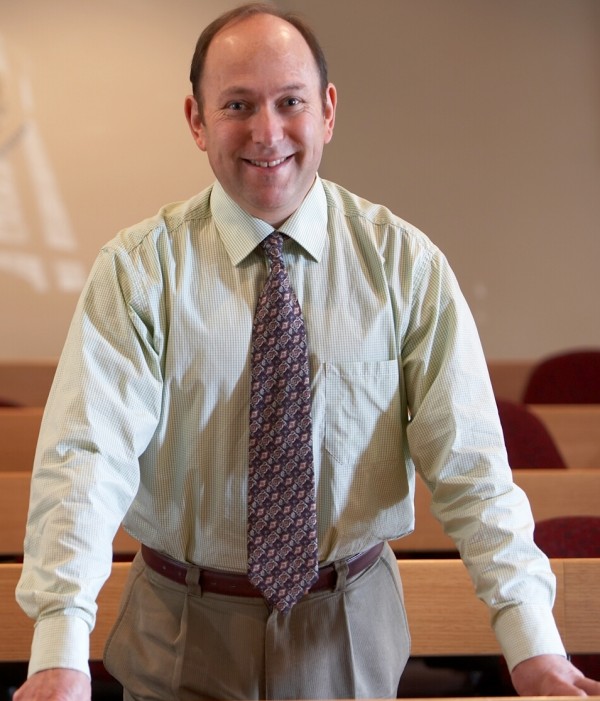
**Michael Berk is Alfred Deakin Professor of Psychiatry in the School of Medicine, Deakin University where he heads the IMPACT Strategic Research Centre.** He is also a Professorial Research Fellow at the University of Melbourne in the Department of Psychiatry, the Centre for Youth Mental Health, Orygen Research Centre, and the Florey Institute for Neuroscience and Mental Health. His predominant interests include risk factors for and prevention of mood disorders, and the discovery and implementation of novel therapies.

**Figure:**
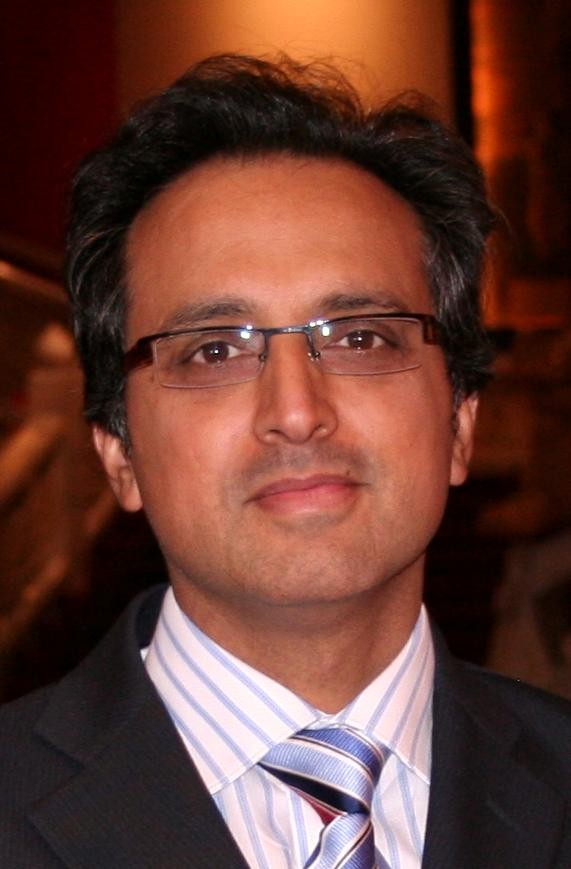
**Gin S Malhi is Head of the Department of Psychiatry, Sydney Medical School, University of Sydney, and also Director of the CADE Clinic, Royal North Shore Hospital.** His research interests include the use of clinical and neuropsychological assessments in conjunction with neurobiological probes to investigate the neural basis of mood disorders.

A welcome change to the criteria for mania is the emphasis on increased activity and energy as well as mood in Criterion A for manic and hypomanic episodes [1]. This is concordant with new research on the primacy of energy clinically, reflecting new data on the role of mitochondrial energy generation in the disorder, as well as the poor specificity of ‘irritability’ [43].

The second major change is ‘an addition’ to bipolar disorder, with broadening of the criteria for mixed states. It has been correctly argued that DSM-IV did not allow for the presence of mixed states in bipolar-II disorder, and convincingly noted that this requirement, which necessitated the rare occurrence of threshold mania and depression, was too restrictive [44]. Reviews of more recent data have supported this view, and led to amendments in DSM-5 that now allows for mixed mania and mixed depression, which are defined by the simultaneous presence of three or more features from the opposite pole of the illness [45]. Some key symptoms that potentially hold diagnostic significance, such as psychomotor agitation, have been excluded on the assumption that they occur in both poles of the illness. Interestingly, discernible treatment and course effects emerge, along with the presentation of opposite polarity symptoms with both depressive and manic episodes, even at relatively low numbers (one or two symptoms). Clinically, the latter modest admixtures of symptoms are remarkably common. However, the threshold of three symptoms has been criticized for being potentially too restrictive [46]. Additionally, the attempt to build a bridge between the constructs of bipolar mixed states and non-bipolar agitated depression highlights the spectral nature of the unipolar-bipolar continuum. The first studies confirming the validity of these criteria have already appeared [47].

Other significant changes include the incorporation of criteria for disruptive mood dysregulation disorder (DMDD), which will influence the field of child psychiatry [48]. This change has occurred in reaction to discordance between putative child and adult bipolar populations with respect to clinical, treatment response, and prognostic factors, and the growing risk of overdiagnosis of bipolar disorder in childhood. However, longitudinal studies suggest that DMDD has poor diagnostic reliability, and cannot be differentiated adequately from oppositional defiant disorder and conduct disorder. Furthermore, it is not associated with current, future-onset, or parental history of mood or anxiety disorders, confirming the utility of the revision [49].

Removal of the bereavement exclusion for a depressive diagnosis is arguably the most contentious ‘loss’ to the field of mood disorders. Early studies suggested that after bereavement, depressive syndromes are common but transient, and do not require treatment. Based on research studies published since 2006, Zisook and colleagues [50] argued persuasively that depression related to bereavement that occurs in the context of bereavement is no different phenomenologically from depression in other contexts, and has similar genetic and familial patterns, comorbidities, course, outcome, and treatment response. Hence, the bereavement exclusion was itself excluded from DSM-5. This has been critiqued [51], and a number of potential advantages and disadvantages that are likely to ensue have been identified. The key argument is that DSM does not meaningfully distinguish between a normal reaction to loss and the threshold for a disorder, and that abandoning the bereavement exclusion risks overdiagnosis of depression and consequent overtreatment, and in this regard, the criteria for major depression are arguably already broad and non-specific. Alternatively, some have argued for a narrowing of the construct and a return to the concepts of endogenous and melancholic depression, suggesting, for example, that greater severity predicts higher treatment response [52,53].

The changes to the mood disorders taxonomy in DSM-5 are important because of the widespread influence of this classification system. As in many other sections of DSM-5, the underpinning of diagnoses by neurobiology remains to be achieved, and thus the diagnoses are largely descriptive and limited by their dependence on phenomenology and grouping of symptoms and signs according to clinically defined cut-offs and associations. Despite these limitations, the revisions meaningfully incorporate recent advances, the system has widespread applicability, and alternate conceptual frameworks such as RDoC do not yet have overt clinical applicability [54].

### Competing interests

MB has received grant or research support from the NIH, Cooperative Research Centre, Simons Autism Foundation, Cancer Council of Victoria, Stanley Medical Research Foundation, MBF, NHMRC, Beyond Blue, Rotary Health, Geelong Medical Research Foundation, Bristol Myers Squibb, Eli Lilly, Glaxo SmithKline, Meat and Livestock Board, Organon, Novartis, Mayne Pharma, Servier and Woolworths; has been a speaker for Astra Zeneca, Bristol Myers Squibb, Eli Lilly, Glaxo SmithKline, Janssen Cilag, Lundbeck, Merck, Pfizer, Sanofi Synthelabo, Servier, Solvay and Wyeth; and has served as a consultant to Astra Zeneca, Bristol Myers Squibb, Eli Lilly, Glaxo SmithKline, Janssen Cilag, Lundbeck Merck, and Servier. GSM has received grant or research support from the National Health and Medical Research Council, NSW Health, AstraZeneca, Eli Lilly & Co, Organon, Pfizer, Servier, and Wyeth; has been a speaker for AstraZeneca, Eli Lilly & Co, Janssen Cilag, Lundbeck, Pfizer, Ranbaxy, Servier, and Wyeth; and has been a consultant for AstraZeneca, Eli Lilly & Co, Janssen Cilag, Lundbeck, and Servier.

### Impact of the DSM-5 on the diagnosis of bipolar and related disorders

Martin Preisig

**Figure:**
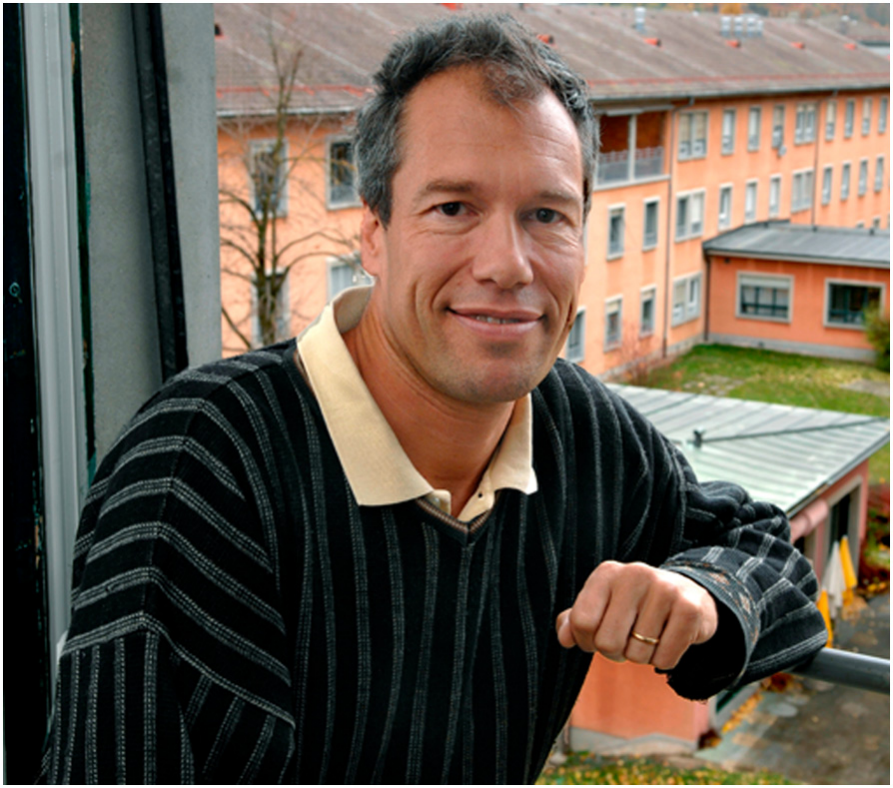
**Martin Preisig is Professor at the Department of Psychiatry of the University Hospital of Lausanne, Switzerland, and head of the Psychiatric Epidemiology and Psychopathology Research Unit.** His main scientific interests include the familial aggregation and genetic determinants of psychiatric disorders, and the association between psychiatric disorders and cardiovascular risk factors.

The DSM-5 [1] includes the following changes to the diagnoses of bipolar and related disorders.

First, Criterion A for manic and hypomanic episodes now requires not only elated, expansive, or irritable mood, but also abnormally and persistently increased goal-directed activity or energy. Although activity and energy are highly relevant features of mania or hypomania, the inclusion of an additional criterion for mania and hypomania raises the diagnostic threshold for the diagnoses of bipolar-I and bipolar-II disorder, and accordingly, it will reduce the number of patients qualifying for these bipolar diagnoses.

Second, antidepressant medication is no longer an absolute exclusion criterion for diagnoses of manic or hypomanic episodes. Indeed, these episodes can now also be diagnosed when they emerge under antidepressant treatment, and when they persist after cessation of this medication. This change is very welcome, as it allows clinicians to also assign the diagnoses of bipolar disorders to the group of patients who develop manic or hypomanic episodes under antidepressant treatment and who otherwise hardly differ from other patients with bipolar disorder.

Third, the diagnosis of ‘bipolar-I disorder, mixed episode’, has been removed. Instead, a new specifier, ‘with mixed features,’ has been added. This specifier applies not only to manic but also to hypomanic episodes when at least three depressive symptoms are present, and to major depressive episodes when at least three manic/hypomanic symptoms are present but the criteria for mania are not met. This is also a very necessary change as it considerably lowers the previous too-high threshold for mixed episodes. However, disorders involving only major depressive episodes will be classified within the category of major depressive disorders, regardless of the presence of mixed episodes, according to this specifier.

Fourth, DSM-5 introduces a new category of other specified bipolar and related disorders. The diagnostic definitions of this category apply to bipolar conditions that do not meet the requirements for bipolar-I or bipolar-II disorders. These conditions include 1) hypomanic episodes of short duration (2 to 3 days) with a history of a major depressive episode, 2) hypomanic episodes with insufficient symptoms and a major depressive episode, 3) hypomanic episodes without prior major depressive episodes, and 4) short-duration (less than 24 months) cyclothymia. This is a big step forward with respect to DSM-IV, which did not include an accurately defined specification of syndromes below the diagnostic level of bipolar-II, although such syndromes have been shown to occur frequently, and should be separated from major depressive disorder.

Finally, for both manic/hypomanic and depressive episodes, a specifier for anxious distress is delineated if at least two anxiety symptoms are present. This new specifier adds another feature to characterize mood episodes more accurately.

In conclusion, the changes to DSM-5 improve the diagnostic classification of bipolar disorders. The introduction of accurate definitions for bipolar conditions below the level of bipolar-II enlarges the array of accurately defined bipolar diagnoses, and should increase clinician awareness of these conditions. Given the currently limited evidence regarding treatment of these conditions, these more accurate definitions will hopefully lead to increased scientific efforts to establish the efficiency of such treatments. Similarly, the more frequent diagnosis of episodes with mixed manic and depressive features that are likely to occur with the DSM-5 changes should also lead to increased interest in bipolar disorders with such episodes, which are in general particularly difficult to treat.

### Competing interests

MP has no competing interests to declare.

## Schizophrenia Spectrum Disorders

### Does DSM-5 facilitate diagnosis of schizophrenia?

Martin Brüne

**Figure:**
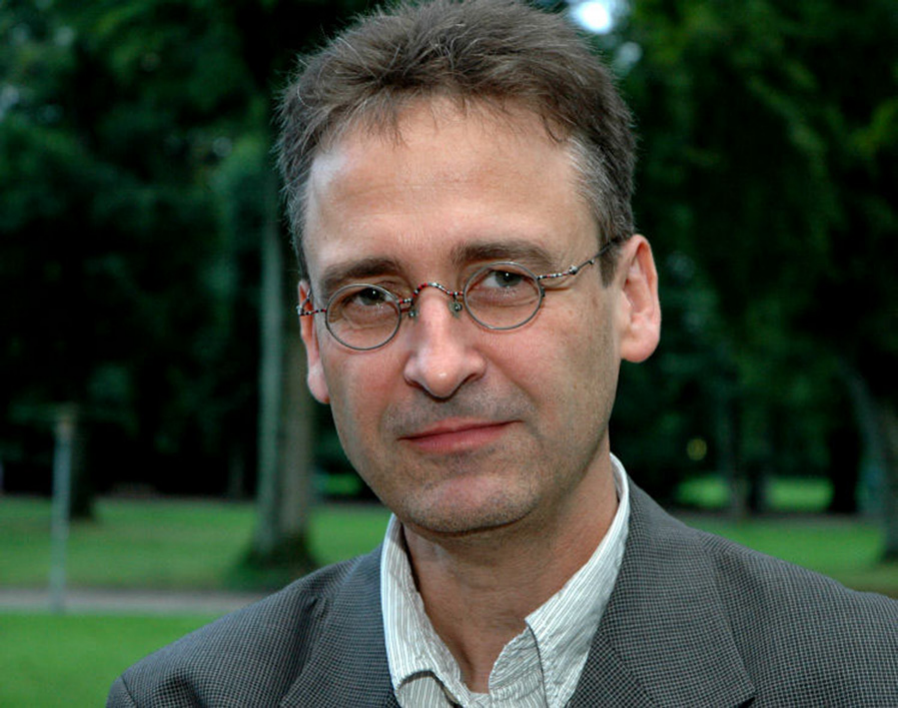
**Martin Brüne is Professor of Cognitive Neuropsychiatry and Psychiatric Preventive Medicine at the LWL University Hospital of Psychiatry, Ruhr-University Bochum, Germany.** His main research interest is social cognition in patients with schizophrenia, and the study of psychopathology from an evolutionary perspective.

In May 2013, the APA released the DSM-5 [1], and even in its nascent state, it has already incited controversy about its usefulness and appropriateness. In this essay, I focus on the clinical issues relating to psychotic disorders, although there are of course other changes made to DSM that are equally debatable. There is also a need for a more general discussion about the usefulness of a categorical classification versus dimensional approaches respecting the applicability of DSM-5 for scientific purposes [55].

With regard to psychosis, the question as to whether or not high-risk states should be included in the manual has been a focus of intense discussion about the pros and cons of doing so. On the one hand, concern has arisen as to whether the inclusion of high-risk states may promote stigmatization of individuals who meet the diagnostic criteria for a high-risk condition, especially in light of findings suggesting that a transition to psychosis occurs in only about 20% of cases within the first 6 months, although this does rise closer to 40% at 3-year follow-up [56]. On the other hand, it has been argued that such an approach could actually improve mental health care by encouraging earlier provision of indicated prevention based on clinical staging, compared with the current clinical reality for most patients with first episodes of psychosis [57]. The inclusion of high-risk states for psychosis in DSM-5 was eventually dropped, possibly owing to recent reports suggesting that early intervention such as cognitive therapy does not reduce the transition rate to psychosis [58], and may at best lead to a delay of psychosis onset [59].

With regard to the diagnostic criteria for schizophrenia, changes include the elimination of the special attribution of bizarre delusions and first-rank auditory hallucinations according to Schneider's criteria. Because Schneider's first-rank symptoms were considered non-specific by the DSM-5 Task Force, and the distinction between bizarre and non-bizarre delusions vague, two Criterion A symptoms are now required in DSM-5 for a diagnosis of schizophrenia. In addition, Criterion A now requires that a patient must meet at least one of three symptoms comprising the presence of delusions, hallucinations, and disorganized speech, which are now regarded as ‘core positive symptoms’. The traditional sub-typing of schizophrenia into paranoid, disorganized (hebephrenic), catatonic, undifferentiated, and residual forms is also now being discarded altogether, based on the view that sub-types are diagnostically unstable, unreliable, and invalid. Instead, a severity rating of the core symptoms has been introduced in recognition of the heterogeneity of the symptomatology.

From a clinician's perspective, it is probably a wise decision not to assign a diagnostic category to high-risk states for psychosis. Although improvement of early detection of early stages of psychosis is still an important mental health issue, there is little evidence to suggest that the inclusion of high-risk states in diagnostic systems can contribute to resolving this clinical imperative. Using a comparison of psychiatry with other medical domains, this would mean that a clinician should include sub-threshold raised blood pressure as a risk factor for hypertension, whereas what actually helps to detect increased risk for hypertension is the regular checking of resting state blood pressure [60]. By analogy, regular mental health checks on a much larger scale than is current in clinical practice may help to discover not only high-risk states of psychosis [61], but also early stages of much more common psychiatric conditions such as depression, anxiety disorder, substance abuse, or dementia. Classification systems can do little about these issues, because they are designed, in the first place, for clinicians to reliably diagnose existing, rather than incipient, conditions of medical (including psychiatric) interest.

With regard to the changes made in DSM-5 respecting the diagnostic criteria for schizophrenia, these seem, at first sight, subtle, but may have more profound effects on how clinicians diagnose and treat patients with schizophrenia. Discarding Schneider’s first-rank symptoms as non-specific breaks with the traditional view that disturbances of ‘ego-boundaries’ (in Anglo-American conceptualizations of psychosis commonly subsumed under the symptomatic spectrum of delusional beliefs) have ‘core’ diagnostic validity. This need not be a bad thing, and it would not be justifiable to keep Schneider’s first-rank symptoms simply based on historical grounds. However, this paradigmatic shift reflects inconsistency of the diagnostic validity of schizophrenia symptoms occurring with high-risk symptoms. In fact, auditory hallucinations in the form of commenting voices and other first-rank symptoms are ‘core’ to all early recognition manuals, including the most widely used Structured Interview for Psychotic Symptoms (SIPS) [62] or the Comprehensive Assessment of At-Risk Mental States (CAARMS) [63]. Put another way, how can a clinician diagnose an at-risk state based on symptoms that are discarded for the full-blown syndrome?

In a similar vein, dropping the traditional sub-types of schizophrenia in DSM-5 based on alleged diagnostic instability over time can be criticized based on evidence from older work suggesting the opposite, especially when rigorous diagnostic criteria apply [64]. The lack of evidence with regard to differences between the sub-types in response to treatment or longitudinal course is probably based more on a lack of research, rather than on a genuine lack of differences in these respects. In fact, it was the explicit goal of the early protagonists in psychiatry such as Kahlbaum and Kraepelin to describe clinical ‘entities’ based on symptomatology (which at the time included not just the subjective aspect of symptomatology, but also non-verbal and paraverbal abnormalities in expression), course and outcome. This approach has certainly failed, at least to some degree, yet many clinicians probably share the view that an insidious early onset of schizophrenia, rapid development of negative symptoms, poor insight, and behavioral abnormalities (that is, hebephrenia) is generally associated with a poorer prognosis respecting recovery, social functioning, and quality of life, compared with patients with acute paranoid schizophrenia.

DSM-5 has been designed to assist clinicians in arriving at reliable diagnoses of psychiatric conditions. Despite the often heated debate about more or less important detail questions, it can be conceded that most changes are minor, with others perhaps being of greater significance. Some diagnoses have been rearranged and reshuffled into new categories, such as OCD, for which, interestingly, a specifier for ‘poor insight’ reflects a dimensional overlap with delusional disorder. Whether or not DSM-5 will change clinical practice very much is a matter for critical evaluation of future prescription practices for both medication and psychotherapy. Personally, I doubt it will.

Akin to naturally evolving bodies, DSM-5 is a ‘design compromise’ between clinical and scientific necessities. It needs to be acknowledged and appreciated that a great deal of scholarly expertise has been spent to further improve diagnostic accuracy for psychiatric conditions. However, what the final product of all these endeavors reflects is that psychiatry has still a long way to go until a reconciliation of divergent perspectives and attitudes about human nature and well-being can be accomplished. In my view, this requires a conceptual integration of the psychological and behavioral adaptations with which *Homo sapiens* is endowed. Such integral perspectives also include the necessity of reconsidering the utility of standard biomedical concepts, such as diathesis-stress models for psychiatric conditions; that is, the evolved biopsychosocial peculiarities that distinguish psychiatric conditions from other medical illnesses [65,66].

### Competing interests

MB has no competing interests to declare.

### Schizophrenia spectrum disorders in DSM-5: In what directions are the most recent changes taking us?

Paul Lysaker

**Figure:**
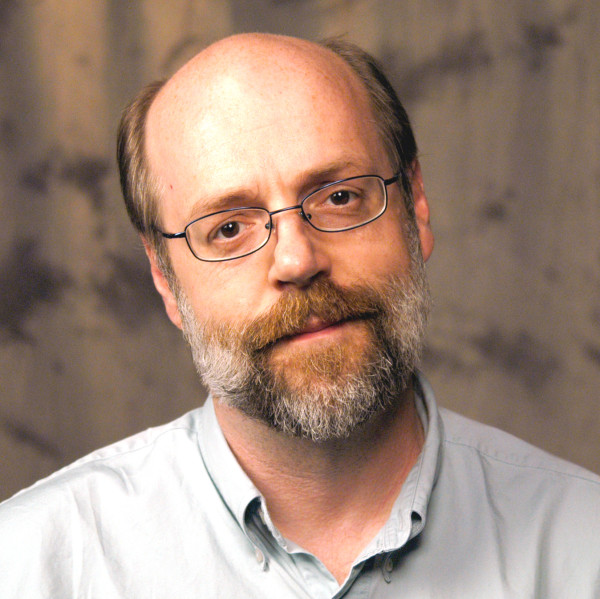
**Paul Lysaker is Professor of Clinical Psychology in Clinical Psychiatry at Indiana University School of Medicine, and Clinical Psychologist and Clinical Supervisor at the Psychiatric Rehabilitation and Recovery Center at the Roudebush VA Medical Center.** His research interests include understanding the roots of psychosocial dysfunction in individuals with schizophrenia, and the development of new rehabilitative and psychotherapeutically based interventions.

The DSM-5 [1], as noted in its own appendix, has made several amendments in its section on Schizophrenia Spectrum and Other Psychotic Disorders. These amendments include the elimination of the older Kraepelinean sub-types such as paranoid or disorganized, and the de-emphasis of bizarre positive symptoms, which previously were on their own sufficient for a diagnosis, but now must be accompanied by at least one other core symptom. Schizoaffective disorder is now presented as a disorder defined more longitudinally, and in section 3, which offers emerging models and measures, a five-point from 0 to 4 scale is presented for clinicians to separately rate the core disturbances of thought, affect, and behavior. Beyond these changes, the supporting text on associated features has been updated to reflect contemporary findings that have overturned long-standing prejudices. Research is noted suggesting the incidence of the disorder men and women is not equivalent as was once widely touted, that random aggressiveness is uncommon, and that prevalence rates do vary across cultures and socioeconomic groups. Although as a whole, the updates appear to match broad surveys of the literature, the new text is not without some puzzling areas in which opinions are presented as fact. We are told that unawareness of illness is equivalent to anosognosia in neurological disorders and that individuals with schizophrenia most often require lifelong ‘living support’ (p. 102) [1]. Arguments exist for both positions, but each is clearly an area of active debate [67-71], and it seems important that these conversations should not be limited with premature claims of consensus.

Of interest in this essay are several questions. Will these changes significantly affect practice? In what direction are these changes taking us? What might be the practical consequences of movement in that direction? The response to the first question seems simple. It appears unlikely the DSM-5 will have any major effect on practice for patients with psychosis. The sub-types and importance of symptoms with bizarre content have not driven current treatment, and their presence previously was possibly a distraction that most clinicians probably ignored. The rating scale presented in section 3 may be useful, just as are any number of existent scales for assessing the psychopathology of individuals with this condition.

Perhaps a more interesting concern, however, relates to the direction in which these changes point. Given what we find now in DSM-5, where are we headed in the next manual and the one after that? Here it strikes me that there is movement in DSM-5 towards more concerted attempts to clearly define the individual components of the disorder but not necessarily to discern how those components are related. It seems possible that the DSM-5 is pointing to future manuals in which there will be increasing efforts to make the pieces of this disorder easier to recognize, but with less concern for core psychopathological processes such as those proposed by Bleuler [72], which allow us to make sense of how schizophrenia spectrum disorders represent a unique set of disorders.

Although the new DSM seems unlikely to change practice, further movement towards a more precise picture of separate components may deeply affect research and treatment efforts. If these more precisely defined elements of the disorder can become targets of very specific treatments, or linked with very specific disturbances in cortical function, then this fragmentation is a cause for optimism. However, given my own reading of emerging research into the complex biological, psychological, social, and political factors that interact in schizophrenia and interrupt the lives of human beings [73], I hold out little hope for the development of a ‘silver bullet’ to treat each fragment of the disorder, or for the discovery of a discrete cause of each fragment. A less optimistic possibility is that the DSM-5 is pointing toward a trend in which we are offered precise accounts of the pieces of schizophrenia that, without an overarching picture of the disorder, exist functionally as fragments. Others have raised concerns that psychiatry and the allied mental health fields are moving back, metaphorically, to a time when medicine treated the fever rather than the infection. In essence, I share this worry. Moreover, I am concerned that if we drift further away from an awareness of the deeper processes at play, we risk losing touch with history and consequently risk becoming more arbitrary. An appreciation of the larger processes possibly at play (for example, loss of the ability to synthesize and build up representations of the self and other [73]) not only allows us to see why schizophrenia as a diagnostic category makes sense, but also assists us to understand and respond to some of the human elements of illness and wellness. It plays a role in keeping alive the view that patients are not merely objects to treat but unique beings who have to make sense of and live with their conditions.

### Competing interests

PL has no competing interests to declare.

## References

[B1] American Psychiatric Association Diagnostic and Statistical Manual of Mental Disorders 2013 5 Arlington, VA: American Psychiatric Association

[B2] American Psychiatric Association Highlights of Changes from DSM-IV-TR to DSM-5 2013 Arlington, VA: American Psychiatric Association

[B3] Friedman MJ Resick PA Bryant RA Brewin CR Considering PTSD for DSM-5 Depress Anxiety 2011 28 750 769 21910184 10.1002/da.20767

[B4] Scheeringa MS Myers L Putnam FW Zeanah CH Diagnosing PTSD in early childhood: an empirical assessment of four approaches J Trauma Stress 2012 25 359 367 22806831 10.1002/jts.21723 PMC6080618

[B5] Cohen JA Deblinger E Mannarino AP Steer RA A multisite, randomized controlled trial for children with sexual abuse-related PTSD symptoms J Am Acad Child Adolesc Psychiatry 2004 43 393 402 15187799 10.1097/00004583-200404000-00005 PMC1201422

[B6] Cohen JA Mannarino AP Iyengar S Community treatment of posttraumatic stress disorder for children exposed to intimate partner violence: a randomized controlled trial Arch Pediatr Adolesc Med 2011 165 16 21 21199975 10.1001/archpediatrics.2010.247

[B7] Lieberman AF Van Horn P Ippen CG Toward evidence-based treatment: child–parent psychotherapy with preschoolers exposed to marital violence J Am Acad Child Adolesc Psychiatry 2005 44 1241 1248 16292115 10.1097/01.chi.0000181047.59702.58

[B8] Lieberman AF Ghosh Ippen C Van Horn P Child–parent psychotherapy: 6-month follow-up of a randomized controlled trial J Am Acad Child Adolesc Psychiatry 2006 45 913 918 16865033 10.1097/01.chi.0000222784.03735.92

[B9] Goldman Fraser J Lloyd SW Murphy RA Crowson MM Casanueva C Zolotor A Coker-Schwimmer M Letourneau K Gilbert A Swinson Evans T Crotty K Viswanathan M Exposure to Trauma: Comparative Effectiveness of Interventions Addressing Maltreatment. Comparative Effectiveness Review No. 89. (Prepared by the RTIUNC Evidence-based Practice Center under Contract No. 290-2007-10056-I.) AHRQ Publication No. 13-EHC002-EF 2013 Rockville, MD: Agency for Healthcare Research and Quality http://effectivehealthcare.ahrq.gov/index.cfm/search-for-guides-reviews-and-reports/?pageaction=displayproduct&productid=1463 23700635

[B10] American Psychiatric Association Diagnostic and Statistical Manual of Mental Disorders (DSM-III) 1980 3 Washington, DC: American Psychiatric Association

[B11] Wessely S Jones E Psychiatry and the lessons of Vietnam: what were they and are they still relevant? War and Society 2004 22 89 103

[B12] Yufik T Simms LJ A meta-analytic investigation of the structure of posttraumatic stress disorder symptoms J Abnorm Psychol 2010 119 764 776 21090877 10.1037/a0020981 PMC4229035

[B13] van Emmerik AA Kamphuis JH Testing a SSM-5 reformulation of posttraumatic stress disorder: Impact on prevalence and comorbidity among treatment-seeking civilian trauma survivors J Trauma Stress 2011 24 213 217 21438018 10.1002/jts.20630

[B14] Stein MB Walker JR Hazen AL Forde DR Full and partial posttraumatic stress disorder: Findings from a community survey Am J Psychiatry 1997 154 1114 1119 9247398 10.1176/ajp.154.8.1114

[B15] Galatzer-Levy I Bryant RA 636,120 ways to have posttraumatic stress disorder: the relative merits of categorical and dimensional approaches to posttraumatic stress Persp Psychol Sciin press 10.1177/1745691613504115 26173229

[B16] Maercker A Brewin C Bryant R Cloitre M Reed G van Ommeren M Humayun A Jones L Kagee A Llosa A Rousseau C Somasundaram D Souza R Suzuki Y Weissbecker I Wessely S First M Saxena S Proposals for mental disorders specifically associated with stress in the ICD-11 Lancet 2013 381 1683 1685 23583019 10.1016/S0140-6736(12)62191-6

[B17] Hollander E Zohar J Sirovataka P Regier D Obsessive-Compulsive Spectrum Disorders: Refining the Research Agenda for DSM-V 2011 Arlington, VA: American Psychiatric Publishing Inc

[B18] Leckman JF Obsessive-compulsive disorder: a review of the diagnostic criteria and possible subtypes and dimensional specifiers for DSM-V Depress Anxiety 2010 27 507 27 20217853 10.1002/da.20669 PMC3974619

[B19] Phillips KA Should an obsessive-compulsive spectrum grouping of disorders be included in DSM-V? Depress Anxiety 2010 27 528 55 20533367 10.1002/da.20705 PMC3985410

[B20] Stein DJ Should OCD be classified as an anxiety disorder in DSM-V? Depress Anxiety 2010 27 495 506 20533366 10.1002/da.20699

[B21] Phillips KA Body dysmorphic disorder: some key issues for DSM-V Depress Anxiety 2010 27 573 91 20533368 10.1002/da.20709 PMC3985412

[B22] Mataix-Cols D Hoarding disorder: a new diagnosis for DSM-V? Depress Anxiety 2010 27 556 572 20336805 10.1002/da.20693

[B23] Stein DJ Trichotillomania (hair pulling disorder), skin picking disorder, and stereotypic movement disorder: toward DSM-V Depress Anxiety 2010 27 611 26 20533371 10.1002/da.20700

[B24] Walkup JT Ferrão Y Leckman JF Stein DJ Singer H Tic disorders: some key issues for DSM-V Depress Anxiety 2010 27 600 10 20533370 10.1002/da.20711

[B25] Feusner JD Phillips KA Stein DJ Olfactory reference syndrome: issues for DSM-V Depress Anxiety 2010 27 592 9 20533369 10.1002/da.20688 PMC4247225

[B26] Bienvenu OJ Is obsessive-compulsive disorder an anxiety disorder, and what, if any, are spectrum conditions? A family study perspective Psychol Med 2012 42 1 13 21733222 10.1017/S0033291711000742 PMC10885736

[B27] Mataix-Cols D Billotti D de la Cruz LF Nordsletten AE The London field trial for hoarding disorder Psychol Med 2013 43 837 47 22883395 10.1017/S0033291712001560

[B28] Lochner C DSM-5 field survey: hair-pulling disorder (trichotillomania) Depress Anxiety 2012 29 1025 31 23124891 10.1002/da.22011

[B29] Lochner C Grant JE Odlaug BL Stein DJ DSM-5 field survey: skin picking disorder Ann Clin PsychiatryJ Am Acad Clin Psychiatrists 2012 24 300 4 23145387

[B30] Stein DJ Phillips KA Patient advocacy and dsm-5 BMC medicine 2013 11 133 23683696 10.1186/1741-7015-11-133 PMC3660192

[B31] Stein DJ Craske MG Friedman MJ Phillips KA Meta-structure issues for the DSM-5: How Do anxiety disorders, obsessive-compulsive and related disorders, post-traumatic disorders, and dissociative disorders Fit together? Curr Psychiatry Rep 2011 13 248 50 21603904 10.1007/s11920-011-0207-1

[B32] Grant JE Skin picking disorder Am J Psychiatry 2012 169 1143 9 23128921 10.1176/appi.ajp.2012.12040508

[B33] Stein DJ What is a mental/psychiatric disorder? From DSM-IV to DSM-V Psychol Med 2010 40 1759 65 20624327 10.1017/S0033291709992261 PMC3101504

[B34] Stein DJ Obsessive-compulsive disorder: diagnostic and treatment issues Psychiatr Clin N Am 2009 32 665 85 10.1016/j.psc.2009.05.007 19716996

[B35] Phillips KA Hart AS Simpson HB Stein DJ Delusional versus nondelusional body dysmorphic disorder: recommendations for DSM-5 CNS Spectrums 2013 1 11 available on CJO2013 23659348 10.1017/S1092852913000266 PMC4948290

[B36] Matsunaga H Symptom structure in Japanese patients with obsessive-compulsive disorder Am J Psychiatry 2008 165 251 3 18006873 10.1176/appi.ajp.2007.07020340

[B37] Mataix-Cols D Pertusa A Leckman JF Issues for DSM-V: how should obsessive-compulsive and related disorders be classified? Am J Psychiatry 2007 164 1313 4 17728412 10.1176/appi.ajp.2007.07040568

[B38] Stein DJ Is disorder X in category or spectrum Y? General considerations and application to the relationship between obsessive-compulsive disorder and anxiety disorders Depress Anxiety 2008 25 330 5 18412059 10.1002/da.20497

[B39] Kendler KS Myers J Zisook S Does bereavement-related major depression differ from major depression associated with other stressful life events? Am J Psychiatry 2008 165 1449 1455 18708488 10.1176/appi.ajp.2008.07111757 PMC2743738

[B40] Blader JC Carlson GA Increased rates of bipolar disorder diagnoses among U.S. child, adolescent, and adult inpatients, 1996–2004 Biol Psychiatry 2007 62 107 114 17306773 10.1016/j.biopsych.2006.11.006 PMC2001259

[B41] Regier DA Narrow WE Clarke DE Kraemer HC Kuramoto SJ Kuhl EA Kupfer DJ DSM-5 field trials in the United States and Canada, Part II: test-retest reliability of selected categorical diagnoses Am J Psychiatry 2013 170 59 70 23111466 10.1176/appi.ajp.2012.12070999

[B42] Wakefield JC DSM-5: proposed changes to depressive disorders Curr Med Res Opin 2012 28 335 343 22201516 10.1185/03007995.2011.653436

[B43] Nierenberg AA Kansky C Brennan BP Shelton RC Perlis R Iosifescu DV Mitochondrial modulators for bipolar disorder: a pathophysiologically informed paradigm for new drug development Aust NZJ Psychiatry 2013 47 26 42 10.1177/0004867412449303 22711881

[B44] Swann AC Lafer B Perugi G Frye MA Bauer M Bahk WM Scott J Ha K Suppes T Bipolar mixed states: an international society for bipolar disorders task force report of symptom structure, course of illness, and diagnosis Am J Psychiatry 2013 170 31 42 doi:10.1176/appi.ajp.2012.12030301 23223893 10.1176/appi.ajp.2012.12030301

[B45] Dodd S Kulkarni J Berk L Ng F Fitzgerald PB de Castella AR Filia S Filia K Montgomery W Kelin K Smith M Brnabic A Berk M A prospective study of the impact of subthreshold mixed states on the 24-month clinical outcomes of bipolar I disorder or schizoaffective disorder J Affect Disord 2010 124 22 8 19944466 10.1016/j.jad.2009.10.027

[B46] Koukopoulos A Sani G DSM-5 criteria for depression with mixed features: a farewell to mixed depression Acta Psychiatr Scand 2013 1 13 doi:10.1111/acps.12140 10.1111/acps.12140 23600771

[B47] McIntyre RS Tohen M Berk M Zhao J Weiller E DSM-5 mixed specifier for manic episodes: evaluating the effect of depressive features on severity and treatment outcome using asenapine clinical trial data J Affect Disord 2013 in press http://dx.doi.org/10.1016/j.jad.2013.04.025i 10.1016/j.jad.2013.04.025 23712026

[B48] Copeland WE Angold A Costello EJ Egger H Prevalence, comorbidity, and correlates of DSM-5 proposed disruptive mood dysregulation disorder Am J Psychiatry 2013 170 173 179 doi:10.1176/appi.ajp.2012.12010132 23377638 10.1176/appi.ajp.2012.12010132 PMC3573525

[B49] Axelson D Findling RL Fristad MA Kowatch RA Youngstrom EA Horwitz SM Arnold LE Frazier TW Ryan N Demeter C Gill MK Hauser-Harrington JC Depew J Kennedy SM Gron BA Rowles BM Birmaher B Examining the proposed disruptive mood dysregulation disorder diagnosis in children in the Longitudinal Assessment of Manic Symptoms study J Clin Psychiatry 2012 73 1342 50 doi:10.4088/JCP.12m07674 23140653 10.4088/JCP.12m07674 PMC3581334

[B50] Zisook S Corruble E Duan N Iglewicz A Karam EG Lanouette N Lebowitz B Pies R Reynolds C Seay K Katherine Shear M Simon N Young IT The bereavement exclusion and DSM-5 Depress Anxiety 2012 29 425 443 22495967 10.1002/da.21927

[B51] Porter R Mulder R Lacey C DSM-5 and the elimination of the major depression bereavement exclusion Aust NZJ Psychiatry 2013 47 391 3 10.1177/0004867413481504 23568160

[B52] Taylor MA Fink M Restoring melancholia in the classification of mood disorders J Affect Disord 2008 105 1 14 17659352 10.1016/j.jad.2007.05.023

[B53] Kocsis JH Melancholia as a distinct mood disorder? Recommendations for DSM-5 Am J Psychiatry 2010 167 1534 21131414 10.1176/appi.ajp.2010.10070983

[B54] Cuthbert BN Insel TR Toward the future of psychiatric diagnosis: the seven pillars of RDoC BMC Med 2013 14 126 23672542 10.1186/1741-7015-11-126 PMC3653747

[B55] Adam D On the spectrum Nature 2013 496 416 418 23619674 10.1038/496416a

[B56] Fusar-Poli P Bonoldi I Yung AR Borgwardt S Kempton MJ Valmaggia L Barale F Caverzasi E McGuire P Predicting psychosis: meta-analysis of transition outcomes in individuals at high clinical risk Arch Gen Psychiatry 2012 69 220 9 22393215 10.1001/archgenpsychiatry.2011.1472

[B57] McGorry PD Hickie IB Yung AR Pantelis C Jackson HJ Clinical staging of psychiatric disorders: a heuristic framework for choosing earlier, safer and more effective interventions Aust NZJ Psychiatry 2006 40 616 22 10.1080/j.1440-1614.2006.01860.x 16866756

[B58] Morrison AP French P Stewart SL Birchwood M Fowler D Gumley AI Jones PB Bentall RP Lewis SW Murray GK Patterson P Brunet K Conroy J Parker S Reilly T Byrne R Davies LM Dunn G Early detection and intervention evaluation for people at risk of psychosis: multisite randomised controlled trial BMJ 2012 344 e2233 22491790 10.1136/bmj.e2233 PMC3320714

[B59] Preti A Cella M Randomized-controlled trials in people at ultra high risk of psychosis: a review of treatment effectiveness Schizophr Res 2010 123 30 6 20727717 10.1016/j.schres.2010.07.026

[B60] American Heart Association Scientific Statement Recommendations for blood pressure measurement in humans and experimental animals. Part 1: blood pressure measurement in humans: a statement for professionals from the subcommittee of professional and public education of the American Heart Association Council on High Blood Pressure Research Hypertension 2005 45 142 161 15611362 10.1161/01.HYP.0000150859.47929.8e

[B61] Kelleher I Harley M Murtagh A Cannon M Are screening instruments valid for psychotic-like experiences? a validation study of screening questions for psychotic-like experiences using in-depth clinical interview Schizophr Bull 2011 37 362 369 19542527 10.1093/schbul/sbp057 PMC3044617

[B62] McGlashan TH Miller TJ Woods SW Pre-onset detection and intervention research in schizophrenia psychoses: current estimates of benefit and risk Schizophr Bull 2001 27 563 70 11824483 10.1093/oxfordjournals.schbul.a006896

[B63] Yung A Phillips LJ McGorry PD Comprehensive Assessment of At-Risk Mental States (CAARMS) 2001 Melbourne: PACE Clinic, University of Melbourne

[B64] Kendler KS Gruenberg AM Tsuang MT Subtype stability in schizophrenia Am J Psychiatry 1985 142 827 832 4014504 10.1176/ajp.142.7.827

[B65] Fabrega H Jr Phylogenetic and Cultural Basis of Mental Illness 2002 New Brunswick: Rutgers University Press

[B66] Brüne M Belsky J Fabrega H Jr Feierman JR Gilbert P Glantz K Polimeni J Price JS Sanjuan J Sullivan R Troisi A Wilson DR The crisis of psychiatry – insights and prospects from evolutionary theory World Psychiatry 2012 11 55 57 22295011 10.1016/j.wpsyc.2012.01.009 PMC3266750

[B67] Kumar A Sharma P Das S Nath K Talukdar U Bhagabati D Insight in psychotic disorder: relation with psychopathology and frontal lobe function Psychopathology In press 10.1159/000348486 23711569

[B68] Lysaker PH Vohs J Hillis JD Kukla M Popolo R Salvatore G Dimaggio G Poor insight in schizophrenia: contributing factors, consequences, and emerging treatment approaches Expert Rev Neurother In press 10.1586/14737175.2013.811150 23898850

[B69] Van der Meer L De Vos AE Stiekema AP Pijnenborg GH Van Tol MJ Nolen WA David AS Aleman A Insight in schizophrenia: involvement of self-reflection networks? Schizophr Bull In press 10.1093/schbul/sbs122 PMC3796073 23104865

[B70] Silverstein SM Bellack AS Scientific agenda for the concept of recovery as it applies to schizophrenia Clin Psychol Rev 2008 28 1108 24 18420322 10.1016/j.cpr.2008.03.004

[B71] Slade M Personal Recovery and Mental Illness: A Guide for Mental Health Professionals 2009 NY, NY: Cambridge University Press

[B72] Bleuler E Dementia Praecox or the Group of Schizophrenias 1950 New York: International Universities Press

[B73] Lysaker PH Bob P Pec O Hamm J Kukla M Vohs J Popolo R Salvatore G Dimaggio G Metacognition as a link which connects brain to behavior in schizophrenia Translational Neurosci In press

